# Female-driven mechanisms, ejaculate size and quality contribute to the lower fertility of *sex-ratio *distorter males in *Drosophila simulans*

**DOI:** 10.1186/1471-2148-8-326

**Published:** 2008-12-02

**Authors:** Caroline Angelard, Catherine Montchamp-Moreau, Dominique Joly

**Affiliations:** 1 Laboratoire Evolution, Génomes et Spéciation, CNRS – UPR 9034 – Avenue de la Terrasse, F - 91 198, Gif-sur-Yvette, Cedex, France; 2 Université Paris-Sud 11, 91 405, Orsay, Cedex, France; 3 Department of Ecology & Evolution, Biophore building, University of Lausanne, CH-1015 Lausanne, Switzerland

## Abstract

**Background:**

*Sex-ratio *meiotic drive refers to the preferential transmission of the X chromosome by XY males. The loss of Y-bearing sperm is caused by an X-linked distorter and results in female-biased progeny. The fertility of *sex-ratio *(SR) males expressing the distorter is usually strongly reduced compared to wild-type males, especially when they are in competition. The aim of this study was to identify the post-copulatory mechanisms that lower the fertility of SR males in *Drosophila simulans*. Parameters contributing to male fertility were measured in single and double mating conditions.

**Results:**

The most detrimental effect on SR males fertility is due to the size of their ejaculate which is half that of wild-type males. Sperm viability and sperm use by the females are also reduced. *Sex-ratio *males are poor sperm competitors in both offence and defence. We found evidence for sperm release from the female reproductive tract that specifically affects SR males. It results in the removal of stored sperm from a first SR mate without the action of the sperm of the second male. In addition, females mated once with an SR male remate more frequently with wild-type males.

**Conclusion:**

The paternity reduction of SR males in competitive conditions is greater than that attributable to their low sperm production and could prevent the spread of distorter X chromosomes in populations when multiple mating occur. The female-driven mechanisms are shown to play a major role both throughout the post-copulatory selective process and increased polyandry. The variation in male reproductive performance may drive the evolution of sexual learning capability of females.

## Background

Male fertility depends on a variety of factors among which the number of sperm in the ejaculate, their quality (such as sperm size, longevity, viability and mobility) and that of seminal products are crucial. In most vertebrates and invertebrates, males that produce larger amounts of sperm increase their reproductive output. Sperm production becomes even more a key factor, but not the only one, when competition for mates is intense and continues within the genital tract of females [1-4]. Mixing of ejaculates from rival males may then occur and promote sperm competition for access to eggs. This includes the spatial displacement of sperm of previous mates (sperm displacement), the non-random utilization of sperm from a particular male (sperm precedence) and the killing or disabling of rival sperm (incapacitation) [5]. However, females are not passive and have evolved several mechanisms that promote fertilization by the sperm of a particular male [6]. Recent research, consistent with the notion that females might select sperm from different mates, highlights a number of sophisticated mechanisms that bias the paternity. Among these mechanisms, the release of sperm or ejaculate of unpreferred males have potentially been underestimated [7,8].

Segregation distorters are selfish genetic elements that manipulate the genome to increase their transmission to the next generation by affecting sperm during spermatogenesis (meiotic drive *sensu lato*) [9]. The known distorters are usually detrimental to male fertility [9]. This harmful effect is expected to promote the evolution of mechanisms that reduce the risk for females to fertilize their eggs by sperm from distorter males. In case of sperm competition, several studies have shown that segregation distorters reduce the competitive ability of males [10-12]. Thus a polyandrous behaviour of females will be favoured because it reduces the reproductive success of males carrying segregation distorters [13].

The *sex-ratio *segregation distortion system is a case of meiotic drive of the X chromosome against the Y chromosome documented in a dozen *Drosophila *species and some other dipterans [14]. It results in a large excess of females in the progeny of *sex-ratio *males (SR males). In *Drosophila simulans*, while X chromosomes carrying a *sex-ratio *distorter (X^SR^) have recently invaded natural populations [15,16], they were unable to invade experimental populations and, on the contrary, were quickly eliminated [17]. These contrasting outcomes seem related to a differential fertility loss of SR males, depending on mating conditions [10]. Whereas they are as fertile as non-driving (standard) males in free-mating conditions with one female per male, they suffer a fertility disadvantage under single mating or in competitive conditions. In the latter case only, the effect on male fertility exceeded the segregation advantage of the X^SR ^chromosome [10]. Thus *sex-ratio *distorters should either spread or disappear from populations, depending on the mating rates of males and females. However the causes of this fertility loss in single matings and in case of sperm competition remain to be identified.

The study of spermatogenesis in *D. simulans *SR males has shown meiotic abnormalities that affect specifically the Y chromosome and include abnormal development of some of the resulting spermatids [18]. The X-bearing spermatids develop normally, and are then expected to be produced in equal quantity by SR and standard males [18,19] unless the process of elimination of Y chromosome sperm has detrimental effects on the X chromosome sperm. So far, the precise fate of the defective spermatids is unknown [18], yet depending on the scenario of mating frequency, the consequences for male fertility can vary. First, they may be discarded during the individualization process [20], thus not giving rise to mature sperm. Second, they may give rise to mature sperm transmitted to the females but released or used less by females. Cases of skew in sperm use depending on male genotype have been reported by Pizzari & Birkhead [21] on feral fowl. Finally, they may result in mature sperm transmitted to the females and eventually stored but defective in fertilization, as it is the case with the *t *haplotype, an autosomal segregation distortion system of the house mouse [22]. In the first case, the quality of the ejaculate should not be affected and the impact on the fertility of SR males is expected to depend on whether the number of sperm produced is a limiting factor in *D. simulans*. In the two other cases, the quality of the ejaculate of SR males should be affected, and so a fertility loss is expected even if sperm is produced in large excess.

The strong disadvantage of *D. simulans *SR males in sperm competition [10] may simply be due to a smaller number of functional sperm transmitted to the females or also involve mechanisms specific to sperm competition, in relation to the quality of the ejaculate and/or its perception by the female. In *Drosophila*, sperm from the second male usually takes precedence over that of the previous mate [23-27], but the mechanism(s) underlying this precedence remain controversial [7,28-30]. It has been proposed that sperm of previous males can be physically displaced from the seminal receptacle and incapacitated (by seminal fluid) [29]. However, Snook and Hosken [7] (whose hypothesis on sperm ageing has recently been criticized [30]), alternatively proposed that females could release all or some stored sperm of their first mate.

In this study we investigated the different post-copulatory mechanisms that may contribute to reduce the fertility of *sex-ratio *males in *D. simulans *and examine the potential consequences on female mating behaviour and fate of X^SR ^chromosome in this species. First, we compared SR and standard (ST) males, regarding the amount of sperm transferred during a single mating, its quality (viability) and also the efficiency of sperm storage and use by the females. Second, we compared the performance in sperm competition of the two types of males and addressed the issue of sperm incapacitation and sperm release by a double mating experiment in which the second male transferred only seminal fluid. The rate and duration of mating during the different experiments were also examined.

## Results

### Percentage and duration of mating

Results are summarized in Table 1. Under first mating conditions, the percentage of copulation was high and similar for SR and ST males (*G*_*adj *_= 2.94, d.f. = 1, *p *= 0.086). Under second mating conditions, when the first mate was an SR male, a second ST male had a higher percentage of copulation compared to a second SR male (*G*_*adj *_= 5.34, d.f. = 1, *p *= 0.021). Moreover, in this case, the mating rate of ST male was the same as under first mating conditions (*G*_*adj *_= 0.11, d.f. = 1, *p *= 0.7) while the mating rate of SR males as second mate was lower than under first mating conditions (*G*_*adj *_= 14.77, d.f. = 1, *p *< 0.001). In contrast, when the first male was an ST male, there was no difference between SR and ST males as second mate (*G*_*adj *_= 1.06, d.f. = 1, *p *= 0.3), but both genotypes showed a copulation rate lower than in first mating conditions (*G*_*adj *_= 60.00, d.f. = 1, *p *< 0.001 for SR males and *G*_*adj *_= 45.09, d.f. = 1, *p *< 0.001 for ST males). The mean mating duration was not significantly different in first and in second mating, independently from genotype and order of both males (Kruskal-Wallis test,χ^2 ^= 8.12, d.f. = 5, *p *= 0.1).

**Table 1:** Percentage and duration of mating.

Crossing	Percentage of mating (%)	Mating duration (min ± SE)	N
*First mating*			
SR	90.00	27.35 ± 0.23	198
ST	84.55	26.33 ± 0.25	186
*Second mating* *(first male/second male)*			
SR/ST	82.81	27.46 ± 1.01	53
ST/SR	42.40	30.33 ± 1.25	18
SR/SR	60.00	29.48 ± 2.36	18
ST/ST	32.56	29.08 ± 4.15	14

*First mating *corresponds to the single mating experiments and to the first mating of the double mating experiments. *Second mating *corresponds to the second mating of the double mating experiments and values are those of the second male. N = number of mating.

### Ejaculate size, sperm storage, viability of sperm and progeny size

**Figure 1:**
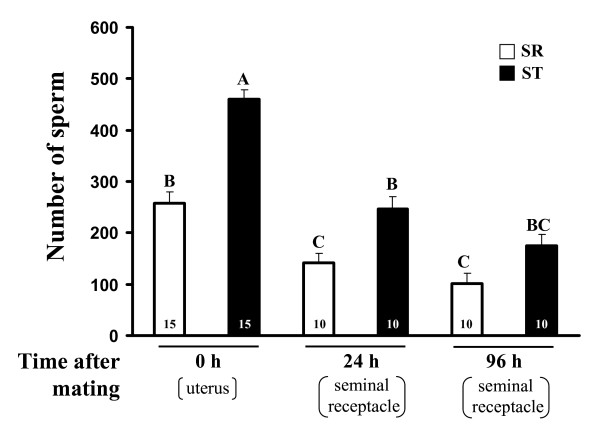
**Number (mean + SE) of transferred sperm (uterus) and stored sperm (seminal receptacle) following a mating**. Different letters above bars indicate a significant difference according to the *Tukey-kramer *HSD test. Numbers inside bars represent sample sizes.

The number of sperm observed in the females' uterus immediately after mating with an SR male was half that of an ST male (Fig. 1; 257.2 ± 22.87 and 459.73 ± 18.11 for SR and ST males, respectively). There was a significant effect of time, male genotype, and their interaction on the number of transferred and stored sperm (Table 2). However, there is no significant difference in the proportion of stored sperm (number of stored sperm at 24 h divided by number of transferred sperm) between SR and ST males (55.1%. and 53.6%, respectively, *G*_*adj *_= 0.14, d.f. = 1, *p *= 0.7).

**Table 2:** Analysis of variance for the number of sperm of an SR or ST male, 0 h, 24 h and 96 h after a mating and for the percentage of dead sperm of an SR or ST male, 24 h and 96 h after a mating.

Source	d.f.	SS	F	*p*
*Number of sperm in single mating*				
Time after mating	2	65621.45	61.84	< 0.001
Male genotype	1	269943.10	50.16	< 0.001
Time after mating × male genotype	2	58484.63	5.43	0.007
				
*Percentage of dead sperm in single mating*				
Time after mating	1	0.256	33.41	< 0.001
Male genotype	1	0.06	8.32	0.007

**Figure 2:**
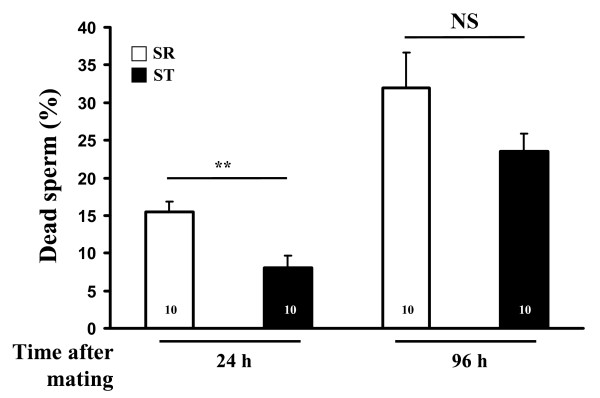
**Percentage (mean + SE) of dead sperm in the seminal receptacle 24 h and 96 h after a mating**. ** *p *= 0.003; NS: *p *= 0.1 (*t*-test). Numbers in bars represent sample sizes.

We observed an additional amount of dead sperm (~8%) for SR males both at 24 h and 96 h after mating (Fig. 2 and Table 2). The proportion of dead sperm increases significantly, but to a similar extent for both types of males (no significant interaction between genotype and time, Table 2).

Following a single mating, SR males produced twice less offspring than ST males (61.60 ± 10.54, N = 10 and 148.60 ± 5.62, N = 10, respectively; *F *= 53.08, d.f. = 1, *p *< 0.001). However, the number of female offspring did not significantly differ (57.8 ± 9.53 and 72.7 ± 3.89, respectively; *F *= 2.09, d.f. = 1, *p *= 0.2) showing that the elimination of Y chromosome sperm does not have any detrimental effect on X chromosome sperm. The percentages of transferred sperm used to produce offspring (number of offspring divided by the number of transferred sperm) were 23.9% and 32.3% for SR and ST males, respectively, while the percentages of stored sperm at 24 h used to produce offspring (number of offspring divided by the number of stored sperm) were 43.5% and 60.3% for SR and ST males, respectively. In both cases, the efficiency was significantly higher for ST males than for SR males (*G*_*adj *_= 5.67, d.f. = 1, *p *= 0.017 for transferred sperm; and *G*_*adj *_= 9.39, d.f. = 1, *p *= 0.002 for stored sperm).

### Sperm precedence

The aim of this experiment was to investigate sperm competitive success of SR and ST males in competition with one another. To do this, females were mated first with an SR male and then with an ST male, and vice-versa.

The size and sex ratio of the progeny produced by the double mated females before and after the second mating are shown in Table 3. The progeny produced before the second mating provided estimates of the proportion of females produced by the ST and SR males in the experimental conditions (52.3 ± 4.1% and 95.3 ± 1.4%, respectively). These were then used to calculate the precedence values: *P*_1SR _= 0.12 and *P*_2ST _= 0.88, and *P*_1ST _= 0.66 and *P*_2SR _= 0.34 (see Methods section). Those values indicate clearly a strong disadvantage of SR males in competitive situations; they sired less offspring in both offence (*P*_1_) and defence (*P*_2_). As a comparison, *P*_2 _= 78% in a control experiment using two ST strains (one of them carrying the visible mutation *scarlet*) under the same conditions as those described above (72 h break between the two matings).

**Table 3:** Weighted mean size ± SE and sex proportion of the progeny produced by double mated females.

Crossing (N)	Male's progeny	Before second mating (% females)	After second mating (% females)	Total progeny (N females)
ST/SR (7)	ST	83 ± 7 (52.3)	70.9*	153.9* (80.5)
	SR	-	36.5*	36.5* (34.8)
	*total*	83 ± 7 (52.3)	107.4 ± 11.2 (66.6)	190.4 ± 15.3

SR/ST (17)	ST	-	89.7*	89.7* (46.9)
	SR	55.2 ± 7.6 (95.3)	12.2*	67.4* (64.2)
	*total*	55.2 ± 7.6 (95.3)	101.9 ± 5.3 (57.3)	157.1 ± 9.4

* calculated from the sex proportions observed before the second mating. N = number of mating.

However, the calculation of precedence considers only the number of offspring sired after the second mating (Table 3), which is the usual procedure. It must be pointed out that a high number of offspring was produced before the second mating (35% and 44% on the total number of progeny for SR/ST and ST/SR respectively, Table 3). Considering the total progeny sired by each male, those sired before and also after the second mating, SR males are still at a disadvantage in both situations. It is worth noting that as second mate, SR males produce on average more female offspring (carrying the X^SR ^chromosome) than the first ST mate. Thus, in this case, the X^SR ^chromosome is better transmitted than the X^ST ^chromosome.

### Sperm incapacitation and sperm release

**Figure 3:**
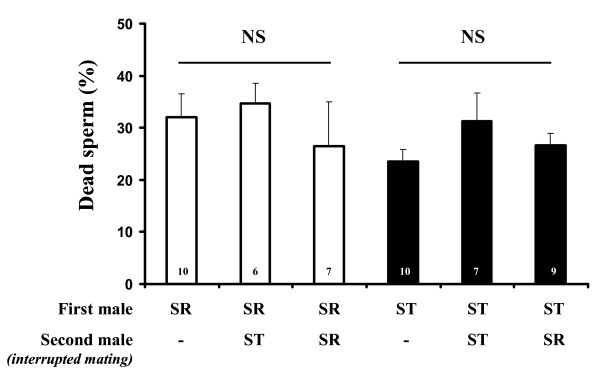
**Percentage (mean + SE) of dead sperm in the seminal receptacle 96 h after a mating, depending on whether a second interrupted mating with only seminal fluid transfer occurred in the interval**. Numbers in bars represent sample sizes. NS = non-significant difference according to the crossed two-way ANOVA.

**Figure 4:**
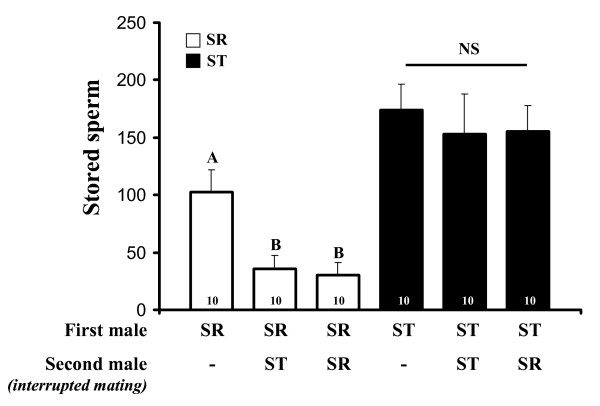
**Number (mean + SE) of sperm stored in the seminal receptacle 96 h after a mating, depending on whether a second interrupted mating with only seminal fluid transfer occurred in the interval**. NS = non-significant difference. Different letters above bars indicate a significant difference according to the *Tukey-kramer *HSD test. Numbers in bars represent sample sizes.

We found that the seminal fluid of a second mate did not increase the proportion of dead sperm of a first mate whatever the genotype of the second mate (Fig. 3 and Table 4). In contrast, we found a strong effect of the first mate's genotype and a non-significant effect of the nature of the second mating (second ST mate, second SR mate or without remating) on the amount of stored sperm (Fig. 4 and Table 4). To test for sperm release following a second mating, we conducted a distinct one-way ANOVA for both first mate genotypes. When the first male was SR, the number of sperm in the seminal receptacle decreased by more than 65% compared to those observed under single mating conditions (*F *= 7.56, d.f. = 2, *p *= 0.002, Fig 4). However, the genotype of the second male did not influence the sperm reduction (Fig 4). Conversely, when the first mate was ST, a second mating did not induce a significant sperm decrease (*F *= 0.18, d.f. = 2, *p *= 0.8).

**Table 4:** Analysis of variance for the sperm incapacitation and sperm release experiments.

Source	d.f.	SS	F	*p*
*Percentage of dead sperm in double mating*				
First male genotype	1	0.022	1.33	0.3
Second interrupted mating	2	0.031	0.92	0.4
				
*Number of stored sperm in double mating*				
First male genotype	1	164431.35	34.66	< 0.001
Second interrupted mating	2	26451.23	2.79	0.070

Comparison of the percentage of dead or stored sperm of a first male (SR or ST) 96 h after a mating, depending on the nature of the second interrupted mating: without second interrupted mating, second SR male, second ST male.

## Discussion

Our study indicates the existence of several mechanisms by which the fertility of SR males is strongly reduced compared to ST males when they compete either directly (double mating) or indirectly (single mating) with ST males. Such a detrimental influence of selfish genetic elements on male fertility is known to be widespread among organisms harbouring them [31,32]. The difference is mainly attributable the size of the ejaculate, which is half that of ST males under single mating conditions, despite similar percentages and durations of the matings. The viability of transferred sperm to the female is also affected, which significantly impacts the efficiency of its use (about a third less). In double mating conditions, we found evidence for a sperm release mechanism that specifically affects SR males and may explain the greater propensity of females to polyandry.

The similar number of females in the progeny of SR and ST males under single mating conditions suggests that the production of fully functional X-bearing sperm by SR males is very similar to that of ST males. This is consistent with previous observations of spermatogenesis in SR males, which suggest that neither the number nor the maturation process of X-bearing spermatids was affected by the *sex-ratio *drive [18,19]. We conclude that the higher rate of sperm death observed in SR males must be attributed to deficient Y-bearing sperm. However, the strongly reduced sperm load of SR males to the females during copulation may be due to the loss of Y-bearing sperm occurring mainly in males. Again, this is consistent with the cytological observation of spermatids resulting from abnormal disjunction of the Y-chromosome that fail to develop properly [18] and are then likely eliminated during the individualization process [20]. Interestingly, the excess of percentage of females in the progeny of *sex-ratio *males has been found to be higher than expected given the mean number of abnormal spermatids per cyst [18]. The present data led us to propose that some sperm, although deficient, are nevertheless passed to the females and contribute increasing the rate of sperm death in the female's storage organs. It can be pointed out that some of the spermatids resulting from abnormal meiosis of the Y chromosome are functional and used to fertilize the eggs, as revealed by the presence of XO males among the progeny [18].

The sperm release phenomenon (i.e. dumping, [7]), which represents an additional load to SR males in multiple mating conditions, can be linked either to first male or second male characteristics or interactions with the female reproductive tract (i.e. female cryptic choice; [6]) or various combinations of those traits. In our study, the differential release observed between driving and non-driving males clearly depends primarily on the properties of their ejaculates as first mate. Indeed, we did not find any effect of the duration of the copulation, nor of the genotype of the second male on the first male sperm release. This led us to propose that the size and the quality of the ejaculate (as the critical parameter for SR males rather than others reproductive features) determine whether the sperm is released. The relationship between sperm release and size of the ejaculate is supported by different reports highlighting the role of female components on sperm precedence [33-36]. In *Odonata*, females can release sperm of unselected males in order to replenish storage organs with sperm of preferred males [37]. In various species, including *Drosophila*, it is known that a low sperm number is a signal that influences females to perform a new copulation [38]. Here we show that mating with driving male promote female polyandry, presumably due to reduced sperm number within the ejaculate. The relationship between remating and sperm release has also been shown in *D. bifurca*, whose females remate more frequently when the sperm of the first male has been released [8]. Our data on mating rates also suggest some kind of learning in the context of sexual behaviour [39,40] that allows females to recognize and preferentially reject unpreferred (SR) males once they have previously experienced an unsatisfactory sperm transfer from a first mate of this genotype. Indeed, while ST and SR males were equally successful under single mating conditions or as second mate when the first mate was ST, SR males were significantly less successful than ST males as second mates when the first male was SR (Table 1). The data of Atlan et al. [10] showed the same tendency but these authors' difference was not significant. In populations of mice harbouring a segregation distorter (*t-complex*) and in stalk-eyed flies carrying a *sex-ratio *distorter, behavioural influence of mate choice has also been detected [41,42].

We found that SR males were at a disadvantage compared to ST males both in offence and defence. Similar patterns were reported in different organisms harbouring such selfish genetic elements [11,12]. Among the three possible mechanisms, i.e. sperm incapacitation, sperm displacement and sperm release, that determine the sperm precedence, the first one cannot explain the lower competitive ability of SR males found in our study (sperm viability is not affected). This contrasts with the situation reported in stalk-eyed flies where the low sperm precedence of SR males depends on incapacitation by seminal fluid from other males [43]. Sperm release, specifically observed when the first mate was SR, can contribute to decreasing *P*_1_. However, we found that in the case of double mating, the total number of offspring of a first SR mate is roughly equal to the number of offspring produce in single mating by this male (Table 3). Consequently, under our experimental conditions, the phenomenon of sperm release and the phenomenon of displacement if it exists, do not contribute significantly to explain the disadvantage of SR males when they are defenders. The low value of *P*_1 _for SR males is then probably mainly the direct consequence of reduced quality and quantity of their ejaculate. A recent study on *sex-ratio *distorter in *D. pseudoobscura *consistently reports that *P*_1 _value of SR males is directly correlated to the lower amount of transferred sperm [12]. However, other factors, such as sperm quality as evidenced in single mating conditions, are likely to play a role in the high reduction of fertility in sperm competition of males carrying driving X-chromosomes; indeed, the paternity reduction is greater than that attributable to the destruction of 50% of the gametes as found in our and other studies [12,32]. Considering the *P*_2 _value, if an SR male has a similar capacity of displacement as an ST male but transfers about half of sperm (57% according to data on Figure 1), we expect a value of *P*_2SR _~0.66 (instead of 0.78 for two ST males). However, the observed value is distinctly lower (0.34). Furthermore, the number of offspring produced by a first ST male (153.9) under double mating conditions is similar to that produced by this male following single mating (148.60 ± 5.62), suggesting that sperm of SR males are incapable of displacing sperm of standard males still present in the female tract. Indeed, neither sperm release nor sperm incapacitation have been detected for a first ST mate, we can thus conclude that the low value of *P*_2 _for SR males is related to their incapacity to displace sperm of a first mate. We hypothesize that the number of sperm transferred by SR males is too low to physically displace previous sperm. Our study stresses the importance of taking into account the number of offspring produced in the interval between two copulations, especially in the case when multiple mating analyses are performed to determine the processes that drive the reproductive success of males. We found that the large majority of SR males' offspring was produced before a second mating with ST males. By adding these offspring and the offspring of SR males produced after the second mating, the X^SR ^chromosome has an advantage of transmission compared to the X^ST ^chromosome. However, this advantage would not have been detected if the offspring sired in the interval would have been ignored.

Compared with the present results, Atlan et al. [10] found a similar disadvantage in defence (*P*_1SR _= 0.10) but a higher disadvantage in offence (*P*_2SR _= 0.51). Since the number of functional sperm produced is expected to be inversely related to the distortion strength (90% in [10] and 95% here), the fact that *P*_2SR _was higher for less distorter males is consistent with our proposal that the sperm displacement ability of a second mate depends on its ejaculate size. While under multiple mating conditions the net result is at disadvantage of X^SR ^over X^ST ^in both studies, our data indicate that when the matings are rare the outcome of the competition could differ, depending on the distortion strength. The level of distortion could therefore have important consequences for the evolution of *sex-ratio *distorters in natural populations. Indeed, if a higher value is associated with stronger deleterious effects, the stronger distorters would not necessarily have a greater advantage and could even be counterselected.

## Conclusion

The reduced size of the ejaculate of *sex-ratio *males, in addition to direct consequences of their fertility, affects the behaviour of females under multiple mating conditions through their sexual learning capability. Our observations lead to the hypothesis that both quality and quantity of the ejaculates may give females the opportunity to select and release sperm of unwanted males in order to perform a new copulation with preferred males. This is in agreement with theoretical predictions of Haig and Bergstrom [13] that selection would favour female behaviour that reduces their exposure as well as that of their offspring to the distorter. This in turn will reduce the frequency of distorters in the populations. While sperm competition is traditionally thought to play a major role in the evolution of polyandry, we show here that counter-balancing female-driven mechanisms deserve further attention.

## Methods

### Strains

**ST8 **is the reference standard stock, which is devoid of distorters and suppressors [44].

**ST8/C(1)RM **is a stock with females carrying the compound X chromosomes C(1)RM, *y*, *w *(from Bloomington stock center) that do not separate at meiosis. These females also carry a Y chromosome. Males are XY as usual and therefore receive the X chromosome from their father and Y chromosome from their mother. This allows the transmission of X chromosome through the paternal lineage. With the exception of the compound Xs, all the chromosomes of this stock come from ST8.

**X^SR6^/C(1)RM **is a line started by crossing a male carrying the *sex-ratio *X^SR6 ^chromosome with ST8/C(1)RM females. The X^SR6 ^chromosome was then maintained in the male lineage by backcrossing males at each generation with ST8/C(1)RM females. More than 50 generations of backcrosses were performed before the beginning of experiments, and so, apart the X^SR6 ^chromosome, this stock is genetically identical to ST8/C(1)RM.

**X^TA107^/C(1)RM **was obtained as the previous line, except that the founder male carried the non driving X^TA107 ^chromosome.

All the flies were reared on axenic standard medium at constant temperature (25°C) under natural photoperiod.

### General mating procedure

Females came from the standard ST8 stock while males from the X^SR6^/C(1)RM and XTA107/C(1)RM lines. For simplicity, they will refer to "SR males" and "ST males", respectively.

The experiments were performed at room temperature in the morning. Flies were sexed upon emergence after a light CO_2 _anesthesia and kept separated by groups of about 10 individuals. For the mating experiments, 3-day old virgin males and females were transferred to individual vials containing medium by aspiration (no anaesthetic). All the matings were visually observed to ensure that full copulation has occurred and that only one mating occurred with a male. The percentage of mating (i.e. the success of copulation) and the mating duration were recorded in each case (at the closest second) and presented in the results section.

### Ejaculate size, sperm storage, viability of sperm and progeny size

These parameters were measured in single mating experiment. One male (either SR or ST) and one female were placed in individual vials and observed during a maximum of 2 h. The male was withdrawn as soon as the mating ended.

A first set of mated females was used to measure the size of the progeny. Each female was allowed to lay eggs for 10 days with a change of vials every 4 days. The total offspring were counted over a period of 15 days.

A second set of females was used to characterize the sperm transmitted by the males. The ejaculate size was estimated as the number of sperm transferred by the male to the uterus of the female. The females were dissected immediately (0 h after the mating) in Phosphate Buffer Saline (PBS) and the number of sperm in uterus was determined by DAPI coloration of nuclei.

A third set of females was used to measure the sperm viability, defined as the percentage of dead sperm in the seminal receptacle of female. It was measured 24 h and 96 h after the mating. This organ is known to be the first source of sperm for fertilization of eggs [28,45,46]. Females were anaesthetized and dissected in PBS. The seminal receptacle was isolated in 8 μl of thyrode buffer and then unrolled. To specifically stain the dead sperm, 0.5 μL of a 1 μM Propidium Iodide (PI) solution was deposited on the slide containing the seminal receptacle which was then incubated for 10 min. Dead sperm, stained in red, were counted under ultraviolet rays with a filter of 630 wave length. Sperm nuclei were then stained with DAPI (which marks all sperm, dead and alive) introduced by capillarity between the slide and the coverslide, which allows to measure the total number of sperm.

### Sperm precedence

After a first mating using the same procedure as above (single mating), the female was reserved for a second mating 72 h afterwards. The two successive males-virgin and 3-day old as in single mating conditions – were of different genotypes (SR then ST and ST then SR). The delay between the two matings was necessary for a substantial proportion of females accepting a second mate during a 4-h period of observation. As soon as the second mating ended, the male was removed, and the female laid eggs during 10 days with a change of vials every 4 days. The total number of offspring was counted over a period of 15 days. Data of the literature and previous results on *sex-ratio *suggest that the rate of male's contribution does not change over time [10]. The proportion of offspring sired respectively by the SR male (*P*_*SR*_) and the ST male (*P*_*ST*_) following the double mating was estimated from the observed proportion of females in the total progeny (*f*) by using the same formula as in Atlanet al. [10]:

*f *= *f*_*ST *_× *P*_*ST*_+ *f*_*SR *_× *P*_*SR*_, where *P*_*SR *_= 1 - *P*_*ST*_

so *P*_*ST *_= (*f*_*SR *_- *f*)/(*f*_*SR *_- *f*_*ST *_)

where *f*_*ST *_and *f*_*SR *_represents the mean percentage of females in the offspring of ST and SR males, respectively, obtained before the second mating (see results section).

*P*_1 _and *P*_2 _are the proportions of offspring sired in double mating by the first and the second mate respectively. The letters added to *P*_1 _and *P*_2 _refer to the genotype of the male, SR for *sex-ratio *and ST for standard males (*P*_1*SR *_and *P*_2*ST *_represent a double mating with first an SR male and then an ST male and *P*_1*ST *_and *P*_2*SR *_for a double mating with first an ST male and then an SR male). Twelve flies (over 220) were eliminated from the dataset, either because they died or did not produce offspring after the first or the second mating. The minimum progeny size following the second mating was 62 flies.

Additionally to this experiment, we determined the percentage of mating and the mating duration in double mating implying two SR males or two ST males. The crossing procedures were the same as above.

### Sperm incapacitation and sperm release

To study the incapacitation and the release of sperm of the first male, we realized double matings with a second male that transferred only seminal fluid. By preventing the sperm displacement of a first male by the sperm of the second male, such a procedure allows detecting sperm release caused by other stimuli associated with the copulation. To do so, we developed a protocol allowing the transfer of seminal fluid from the second mate but preventing the transfer of its sperm, both processes arising successively in *Drosophila *[47]. While it is likely that some seminal fluid is also transferred at the same time as sperm, various papers have already shown a major effect of the seminal fluid transferred before the sperm [48-50]. We then determined the moment of the mating from which male transferred sperm by interrupting it at regular intervals of time. Mating was interrupted by shaking the vial containing the pair. Effective sperm transfer was detected from 15 min of mating by the presence of larvae in vials 3 days later. At 10 min, females did not produce larvae and no sperm was found in the reproductive tracts (n = 24). The crossing procedure was the same as described above except that the second mating was stopped at 10 min.

To look for sperm incapacitation, we compared the percentage of dead sperm 96 h after a single mating with that observed after the same delay when a second interrupted mating had occurred meanwhile. The procedure to measure the percentage of dead sperm in the seminal receptacle of females after a second interrupted mating was the same as for single mating (see above). To look for sperm release, we compared the number of stored sperm 96 h after a single mating with the number of stored sperm after the same delay when a second interrupted mating had occurred.

### Statistics

Data were tested for normality and homoscedasticity before performing ANOVA. For differences in sperm storage efficiency between SR and ST we used a crossed two-way ANOVA with meiotic drive genotype of male and time after mating as factors. For differences in number of offspring produced after single mating, we used a one-way ANOVA. We tested for differences in sperm release and sperm incapacitation between SR and ST males using a crossed two-way ANOVA with genotype of the first male and the nature of the second mating (second ST mate, second SR mate or without second mating) as factors. We compared the percentage of dead sperm 24 h and 96 h after a single mating for SR and ST males using a crossed two-way ANOVA with male genotype and time after the mating as the two factors and we performed *t-tests *to compare the results between males at each time. For the sperm release experiment and the sperm storage efficiency experiment, we carried-out a multiple comparison test using *Tukey-kramer *HSD (honestly significant difference) test with an alpha level of 0.05. Interaction terms were examined when possible and removed from the model when they were not significant. The comparisons of percentages were done by using a *G*-test of independence with a Williams's correction [51]. Descriptive statistics are expressed as mean ± standard errors (SE). Tests were carried out using JMP^® ^software version 5.0 (SAS Institute Inc., 2002).

## Authors' contributions

CA designed, carried out the experiment and analysed the data. CMM and DJ designed and supervised the experiment. The three authors wrote the paper.
